# Microureteroscopy in Children: Two First Cases

**DOI:** 10.1089/cren.2016.0005

**Published:** 2016-03-01

**Authors:** Juan-Pablo Caballero-Romeu, Alberto Budia-Alba, Juan-Antonio Galan-Llopis, Maria-Dolores Montoya-Lirola, Pedro-José García-Tabar, Juan-Francisco Galiano-Baena, Nuria Albertos-Mira-Marcelí, Jeronimo Gonzalvez-Piñera

**Affiliations:** ^1^ Department of Urology, University General Hospital of Alicante, Alicante, Spain.; ^2^ Department of Urology, “La Fe” Polytechnic University Hospital in Valencia, Valencia, Spain.; ^3^ Department of Urology, University Hospital of Vinalopo, Elche, Spain.; ^4^ Department of Pediatric Surgery, University General Hospital of Alicante, Alicante, Spain.

## Abstract

***Background:*** Urinary stones disease is becoming more common not only in adults but also in children. Most cases are resolved with extracorporeal shock wave lithotripsy, but miniaturization of endoscopes has increased the use of ureteroscopy in resolving ureteral stones, most notably in children.

***Case Presentation:*** This presentation focuses on two cases of microureteroscopy. In both cases, the presence of lithiasis in the pelvic ureter was suspected to be the cause of ureter hydronephrosis, and a microureteroscopy was performed for treatment purposes. MicroPerc set 4.85F sheath was used to explore the pelvic ureter, thus avoiding the need to dilate the ureteral meatus or having to use the safety guide. Patients did not require a postoperative stent and were discharged within 24 hours of the procedure.

***Conclusion:*** Use of microureteroscopy proved satisfactory in the two cases of children and it allows diagnosis and treatment of ureteral pathology in pediatric patients.

## Introduction and Background

Urolithiasis is uncommon in children. When urolithiasis does appear, it has a positive response to treatment with extracorporeal shock wave lithotripsy and children can eliminate large size fragments. However, ureteroscopy is a technique to be resorted to in cases of stones in the lower ureter when lithiasis is not expelled within an acceptable time frame.

In many centers, equipment employed for adult patients is also used to handle lithiasis in children, which implies that prior dilation of the meatus is necessary to gain ureteral access, whereas other centers have ureteroscopes designed for children.

Miniaturization of the equipment for percutaneous renal surgery^1^ allows for retrograde treatment^2^ of ureteral lithiasis, which reduces pain, ureteral damage, and the use of catheters after the operation. It also allows for a faster recovery.

In this context, it was decided to use the full length of the 4.8F sheath of the MicroPerc device to diagnose and treat ureteral pathologies in children.

## Clinical Cases

### Case No. 1

A 10-year-old boy was referred to “La Fe” Polytechnic University Hospital in Valencia in December 2014 because of a right ureteral lithiasis. He was completely asymptomatic.

The most relevant pathological incidents occurred at the age of 1 when he was effectively treated for right vesicoureteral reflux through a Deflux right perimeatic submucosal injection. At age 4, he had a left nephrectomy due to a multicystic disorder.

**FIG. 1.**
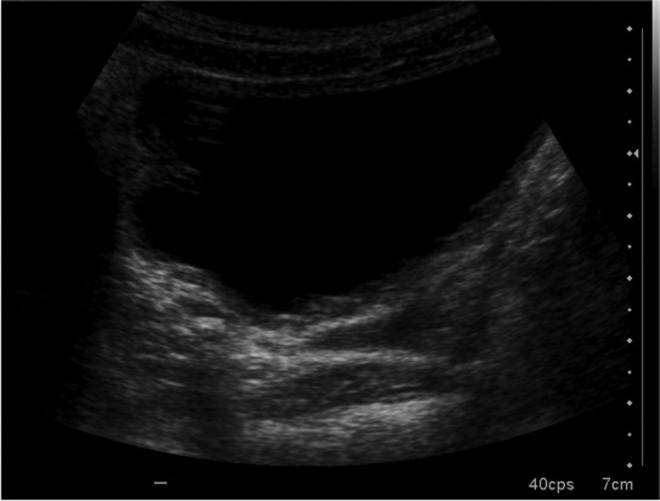
Ultrasonography in which a hyperechoic area is detected on the right distal ureter.

During a routine checkup using ultrasonography, a right ureteral pyelocaliceal ectasia was identified (Fig. 1), which had already been seen in earlier checkups. Inside the dilated right ureter, an echogenic focus with shadowing was identified. KUB showed a calcium density, which measured 4.2 × 4.4 mm in the right pelvic area. This was not seen in earlier checkups. Microureteroscopy was performed using a MicroPerc set 4.8F sheath, optic diameter of 0.9 mm, and standard gravity irrigation.

The bladder showed no significant alteration. The sheath was introduced through the ureteral meatus and no safety guide or dilation balloon was necessary. The ureter was explored up to the radiological level of the image suspicious of lithiasis, but it was not found. After the introduction of a 4.85F sheath in a recess of the bladder mucosal, the injected material for the endoscopic treatment of vesicoureteral reflux was detected.

The duration of the operation was 50 minutes. Placement of a ureteral catheter was not necessary. The patient was discharged in 24 hours without any postoperative complication.

### Case No. 2

An 8-year-old boy went to Alicante University General Hospital emergency center because of pain on his right side and vomiting.

Exploration did not reveal significant problems.

An ultrasonography of the abdomen revealed enlargement of the right kidney associated with increased echogenicity and moderate pyelocaliceal ectasia along the whole tract from the ureter to the distal ureter, where a 7 mm hyperechogenic image was revealed—the latter being compatible with lithiasis.

Pain was treated with intravenous analgesics and was handled conservatively, but the lithiasis was not expelled 8 days after admission to the hospital, and a microureteroscopy was performed using a MicroPerc set 4.85F sheath.

**FIG. 2.**
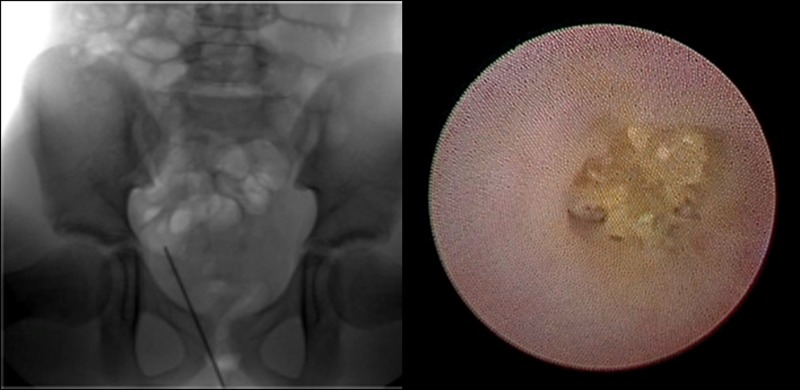
Intraoperative radioscopy in which the sheath for microureteroscopy is identified inside the right ureter and endoscopic vision during the procedure before starting dusting of the stone.

After approaching the ureteral meatus, microuterosocopy was performed without placing a safety guide or dilating the meatus. The ureteroscopy began with a simple and virtually atraumatic passage through the meatus and intramural ureter. An impacted lithiasis was noted at the level of the pelvic ureter (Fig. 2).

A 200-μm laser fiber and an IMEX Acu-H2 40W generator at 6 Hz frequency and a power of 0.6 J were used. The lithiasis was fragmented to less than 200 μm that spontaneously exited toward the bladder during the procedure. At the end of the procedure, the ureter showed a moderate edema at the site of stone impaction. The surgical time was 23 minutes. No ureteral postoperative catheter was used. The patient was discharged 24 hours after the operation.

## Discussion

Lithiasis is a less frequent pathology in children than in adults, but it is endemic in some parts of the world. Extracorporeal shock wave lithotripsy is the treatment chosen for stones less than 2 cm at the kidney and upper ureter levels. In the lower ureter the technique chosen is ureteroscopy.

There is a variability in endoscopic equipment used in children, including semirigid 8F ureteroscopes to telescoped ureteroscopes of 4.5F to 6F (see Table 1).^3,4^

**Table 1.** Series of Ureteroscopies in Pediatric Patients

*Author*	*Year*	*Number of patients*	*Number of procedures*	*Age years old [range]*	*Scope(s)*	*Dilation/preoperative stent*	*Safety wire (%)*	*Postoperative stent (%)*	*Stone-free rate (%)*	*Botched surgery (%)*
A.C. Koura^3^	2007	20	22	5.2 [3–9]	6F/7.5F semirigid (85%) and 8F flexible (15%)	Dilation 100%	100	30	90	10
M.C. Smaldone^4^	2007	100	115	13.2 ± 5.4	7.5F semirigid and/or 6.9F flexible	Preoperative stent 49%, Dilation 66%	100	78	91	0
J.C. Thomas^5^	2005	29	33	7.83 [0.41–12]	7.5F semirrigid or flexible	Dilation 27.6%	>50	93	88	Not available
A.H. Tan^6^	2005	23	27	9.1 [1.5–14]	Not available	Dilation 17.4%	Not available	91	95.20	Not available
C. Kocaoglu^7^	2014	36	38	5.3 [±3]	4.5–6F	0%	100	42.10	97.4	0
A. El-Assmy^8^	2006	32	35	8.7 [2–15]	8F	Dilation 28.56%, Nephrostomy 11.4%	100	97.10	90.70	18
E. Minevich^9^	2005	58	65	7.5	6.9F semirigid or 7F flexible	Dilation 30%	100	85	98	Not available

**FIG. 3.**
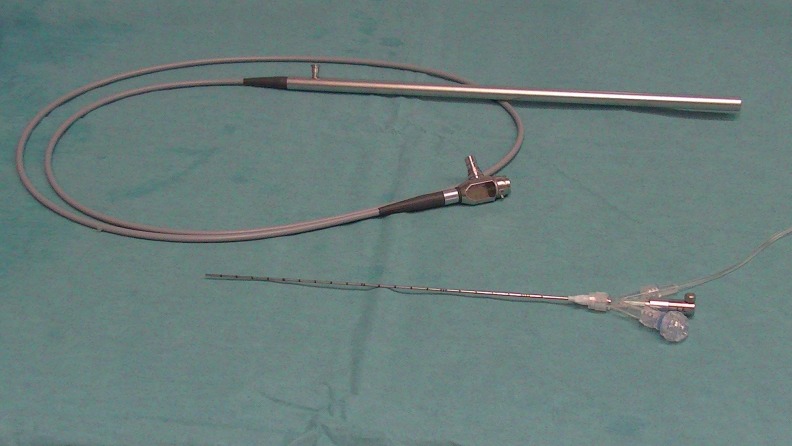
Equipment used in microureteroscopy: 0.9 mm diameter optic, 4.85F sheath of MicroPerc set.

We considered of interest when effected through the Microperc set 4.85F sheath (Fig. 3) thus avoiding the effect of progressive diameter increase and the obstruction of the ureteral meatus.

Image quality obtained and irrigation flow were satisfactory. Irrigation serum was passed around the sheath, thus avoiding high pressure and reducing the associated likelihood of bacteremia. The percentages of ureteral meatus dilation range from 0% to 100% depending on the authors. Whereas use of the safety guide ranges between 50% and 100% of the cases. The need to dilate the meatus in children by using this sheath might depend on the experience of each group. When we perform a conventional ureterosocopy in adults, a safety guide is always used for virtually all the procedures. However, a safety guide was only used in the first case in which a microureteroscopy was performed on an adult woman. In the rest of surgeries the need for its use has not been found to date, in view of the distance of the sheath from the wall of the ureter. Evidently we can employ a safety guidewire if considered necessary.

Postoperative use of the ureteral catheter ranges from 30% to 93% in recent series of procedures on children. In neither of the two cases referred to above was its use necessary, and no additional pain was noted nor was ureteral stenosis noted after 2 months of evolution.

The MicroPerc equipment has a higher initial cost than that of a conventional ureteroscopy; however, this system could reduce the use of dilation balloons, safety guides, ureteral catheters, second admittance into hospital to remove catheters, pain killers, and recovery time. The global cost of the procedure could therefore be reduced with lower morbidity rates.

## Conclusions

Use of microureteroscopy proved satisfactory in the two cases for children, which have arisen to date. Its use allows diagnosis and treatment of ureteral pathology in pediatric patients. Further comparative prospective studies should be performed to determine the usefulness of this new technique and its cost/efficiency ratio.

## Abbreviation Used

KUB: kidney, ureter, bladder X-ray
